# Selective C–H Bond Cleavage with a High-Spin Fe^IV^–Oxido Complex

**DOI:** 10.3390/molecules28124755

**Published:** 2023-06-14

**Authors:** Chen Sun, Jennifer L. Jaimes, Alec H. Follmer, Joseph W. Ziller, Andrew S. Borovik

**Affiliations:** Department of Chemistry, University of California-Irvine, Irvine, CA 92697, USAafollmer@uci.edu (A.H.F.);

**Keywords:** metal-oxido, C-H bond cleavage, Fe(IV)-oxido complexes

## Abstract

Non-heme Fe monooxygenases activate C–H bonds using intermediates with high-spin Fe^IV^–oxido centers. To mimic these sites, a new tripodal ligand [pop]^3−^ was prepared that contains three phosphoryl amido groups that are capable of stabilizing metal centers in high oxidation states. The ligand was used to generate [Fe^IV^pop(O)]^−^, a new Fe^IV^–oxido complex with an *S* = 2 spin ground state. Spectroscopic measurements, which included low-temperature absorption and electron paramagnetic resonance spectroscopy, supported the assignment of a high-spin Fe^IV^ center. The complex showed reactivity with benzyl alcohol as the external substrate but not with related compounds (e.g., ethyl benzene and benzyl methyl ether), suggesting the possibility that hydrogen bonding interaction(s) between the substrate and [Fe^IV^pop(O)]^−^ was necessary for reactivity. These results exemplify the potential role of the secondary coordination sphere in metal-mediated processes.

## 1. Introduction

High-spin *S* = 2 Fe^IV^–oxido species are important intermediates in the catalytic cycles of non-heme iron enzymes capable of hydroxylating inert C–H bonds [1,2,3,4]. Many Fe^IV^–oxido complexes have been synthesized and characterized to further understand the reactivity of non-heme iron enzymes, though most of these complexes have an *S* = 1 spin ground state [2,5,6,7,8,9]. The few examples of synthetic Fe^IV^–oxido complexes that are *S* = 2 [10,11,12,13] are generally more reactive than their *S* = 1 counterparts [1]; however, the mechanistic details, including the role of spin states, are still emerging. Moreover, it is often difficult to make comparisons between different biomimetic Fe^IV^–oxido complexes because most are not derived from the same ligand scaffold, which can influence different factors that govern reactivity.

**Figure 1:**
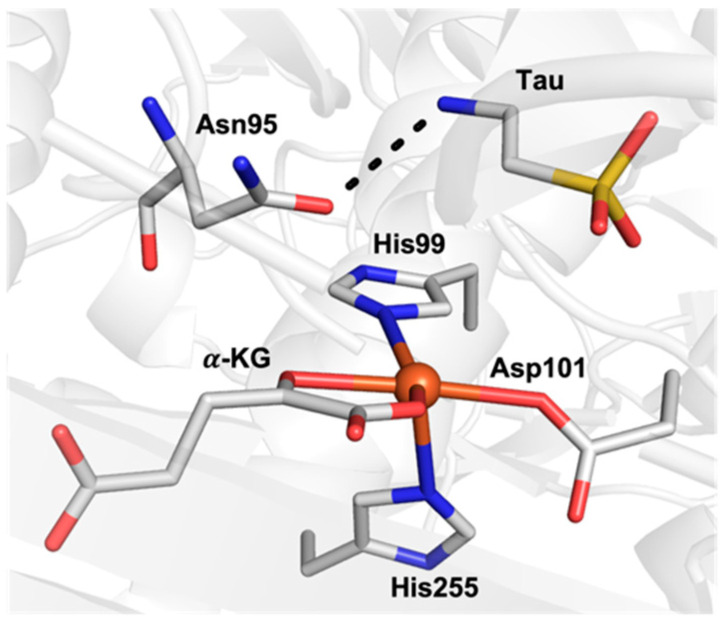
Active site of TauD showing the substrate tau H-bonded to Asn95 (PDB: 1OS7).

Properties other than the spin state can also impact the function of Fe^IV^–oxido species; for instance, we and others have argued that the pKa of the oxido ligand can have a large influence on C–H bond cleavage via proton-dominated PCET processes [14]. Furthermore, interactions within the secondary coordination sphere, such as hydrogen bonds (H-bonds) can impact the electronic and structural properties of Fe^IV^–oxido species [10,15]. For example, the *α*-ketoglutarate (*α*-KG)-dependent enzyme, taurine dioxygenase (TauD), utilizes a reactive *S* = 2 Fe^IV^–oxido intermediate to hydroxylate its substrate, taurine (tau) [4,16]. Results from X-ray crystallographic studies on TauD revealed that tau is anchored in close proximity to the active iron center through an H-bonding interaction with a nearby amino acid residue (Asn95; Figure 1) [17,18]. This H-bond is suggested to have important implications in the positioning of tau near the iron center, which leads to enzymatic function. Within a broad context, using H-bonds to position and orient substrates around active sites of proteins is recognized as playing a role in substrate functionalization [19]. Thus, our group sought to understand how H-bonds installed within the secondary coordination sphere might govern how a metal complex reacts with external substrates.

**Figure 2:**
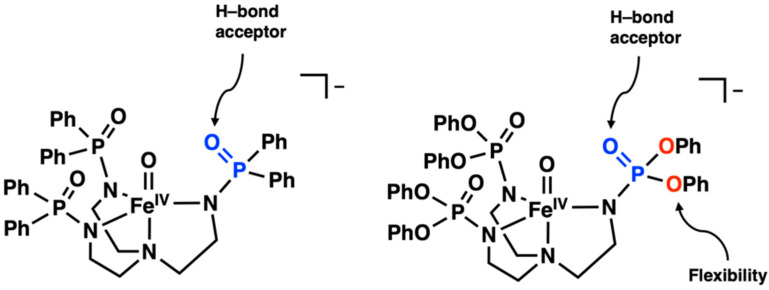
Structures of [Fe^IV^poat(O)]^−^ (**left**) and [Fe^IV^pop(O)]^−^ (**right**).

Recently, our group introduced the tripodal ligand [poat]^3−^ (*N*,*N*′,*N*″-[nitrilotris-(ethane-2,1-diyl)]tris(*P*,*P*-diphenylphosphinic amido), which contains phosphinic amido groups that can stabilize high-valent Fe centers, such as the high-spin *S* = 2 Fe^IV^–oxido complex, [Fe^IV^poat(O)]^−^ (Figure 2, left) [20]. However, [Fe^IV^poat(O)]^−^ was found to be unreactive toward the cleavage of C–H bonds in most external substrates [21], which we proposed was caused by the phenyl rings within the [poat]^3−^ backbone, limiting access to the Fe^IV^–oxido unit. To address this issue, we designed [pop]^3–^, which instead contains phosphoryl amido groups, and prepared the corresponding Fe^IV^–oxido complex, [Fe^IV^pop(O)]^−^ (Figure 2, right). As shown, both [poat]^3−^ and [pop]^3–^ produce nearly the same primary coordination sphere composed of a trianionic ligand field, which supports the formation of high-valent metal centers. In addition, both ligands contain a secondary coordination sphere composed of P=O groups, which can serve as H-bond acceptors to help stabilize reactive species generated during a reaction [22]. However, unlike [poat]^3−^, the [pop]^3–^ ligand contains an ether linkage between the P=O groups and the phenyl rings. This difference in structure is proposed to provide more flexibility within the ligand framework to create a more exposed metal site, which, in turn, could promote reactivity with external substrates by allowing more accessibility. In this study, we describe the preparation and spectroscopic characterization of [Fe^IV^pop(O)]^−^ and its ability to cleave C–H bonds. Our studies indicate that reactivity can be achieved only with substrates containing a functional group that can possibly H-bond with the [pop]^3–^ ligand.

## 2. Results and Discussion

**Figure 3:**
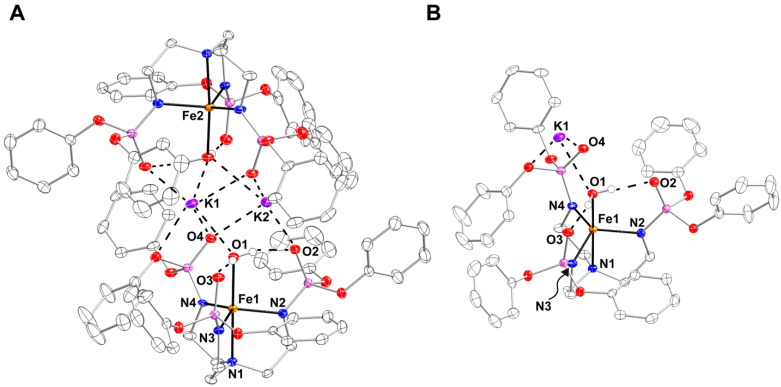
(**A**) X-ray crystal structure of {K[Fe^II^pop(OH_2_)]}_2_ shown as an aggregate, and (**B**) asymmetric unit of K[Fe^II^pop(OH_2_)]. Ellipsoids were drawn at a 50% probability level. All hydrogen atoms were omitted except for those on the aqua ligand for clarity.

**Preparation and Properties of K[Fe^II^pop(OH_2_)].** K[Fe^II^pop(OH_2_)] was used as the starting synthon for the generation of the corresponding Fe^IV^–oxido complex, [Fe^IV^pop(O)]. K[Fe^II^pop(OH_2_)] was generated via the deprotonation of a solution of H_3_pop in anhydrous tetrahydrofuran (THF) with three equiv. of potassium hydride (KH), which was allowed to stir until H_2_ evolution ceased. The resultant mixture was treated with 1 equiv. of Fe^II^(OAc)_2_, allowed to stir for at least 1 h, and then 1 equiv. of degassed H_2_O was added. The reaction mixture was filtered through a medium porosity frit and the filtrate was layered under pentane to yield single crystals (>70%). The molecular structure of K[Fe^II^pop(OH_2_)] was investigated using X-ray diffraction (XRD) methods (Figure 3), which revealed that K[Fe^II^pop(OH_2_)] crystallizes as an aggregate, {K[Fe^II^pop(OH_2_)]}_2_, in the *P*$\overset{–}{1}$ space group (Figure 3A). Similar to complexes in the [poat]^3−^ ligand framework, the K^+^ ion interacts with the P=O groups of [pop]^3–^. The two [Fe^II^pop(OH_2_)]^−^ complexes are linked through two K^+^ ions via the O-atoms of the phosphoryl amido arms and the aqua ligands that are bound to the Fe^II^ centers in an axial coordination site. Each Fe^II^ center is five-coordinate with a trigonal bipyramidal (TPB) geometry. The Fe1–N1 and average Fe1–N_equatorial_ bond lengths are 2.214(2) and 2.063(2) Å, respectively, and the average N1–Fe1–N_equatorial_ angle is 81.25(6)°. The Fe^II^ center is displaced 0.313 Å from the equatorial plane toward the aqua ligand with an Fe1–O1 bond length of 2.205(1) Å, which is relatively longer than in other reported Fe^II^–aqua complexes in TPB geometry [23,24]. This elongation could possibly be due to the interaction between the K^+^ ions and the O-atom of the aqua ligand, which should weaken the Fe1–O1 bond. The molecular structure also exhibits two intramolecular H–bonds between the aqua ligand and the P=O groups of two of the three phosphoryl amido arms, with O1•••O2 and O1•••O3 distances of 2.649(2) and 2.728(2) Å, respectively, demonstrating the utility of the P=O groups as H–bond acceptors (Figure 3B).

**Scheme 1:**
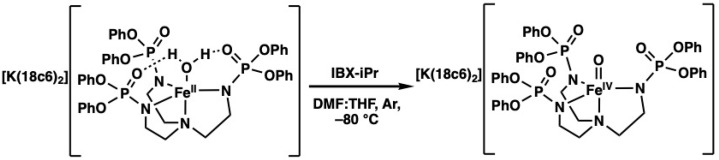
Generation of [Fe^IV^pop(O)]^−^ in 1:1 DMF:THF at −80 °C.

**Figure 4:**
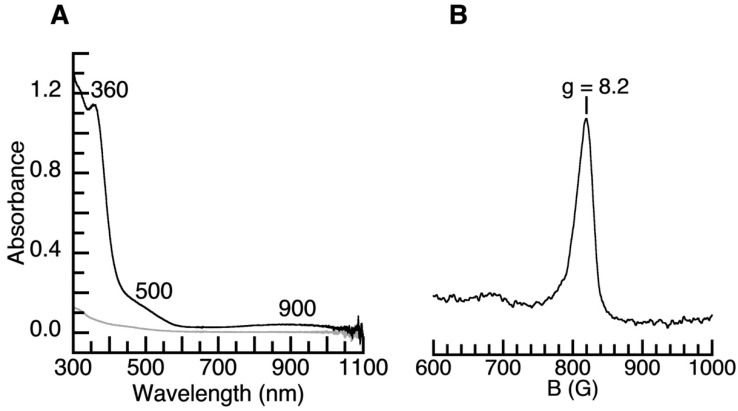
(**A**) Electronic absorption spectrum of [Fe^IV^pop(O)]^−^ generated from the reaction of 0.39 mM of K(18c6)_2_[Fe^II^pop] + 1.05 equiv. of IBX-iPr in DMF:THF at −80 °C. (**B**) The parallel mode EPR spectrum of [Fe^IV^pop(O)]^−^ collected at 12.3 K in DMF:THF at −80 °C.

**Preparation and Properties of [Fe^IV^pop(O)]^−^.** [Fe^IV^pop(O)]^−^ was generated in situ by allowing a mixture of K[Fe^II^pop(OH_2_)] and ≥ 2 equiv. of 18-crown-6 (18c6) in an anhydrous 1:1 mixture of *N*,*N*-dimethyl-formamide (DMF) and tetrahydrofuran (THF) at −80 °C to react with 1 equiv. of isopropyl 2-iodoxybenzoate (IBX-iPr) (Scheme 1). In our previous work with [poat]^3−^, it was found that two equiv. of 18c6 is necessary for aiding in solubility and to fully encapsulate the K^+^ ions, which prevents interactions with the P=O groups [20]. The electronic absorption spectrum of the resulting [K(18c6)_2_][Fe^IV^pop(O)] salt (Figure 4A) shows a high-energy feature at λ_max_ = 360 nm, a shoulder at λ_max_ = 500 nm, and a low-energy feature at λ_max_ = 900 nm (see Figure S1, Supplementary Materials), which is attributed to *d–d* transitions that are found in nearly all synthetic Fe^IV^–oxido complexes [1,6,11,12,20]. There is also a feature at 810 nm that is attributed to a Fano interference, which arises from spin–orbit coupling between spin-forbidden and spin-allowed transitions and is also seen in [Fe^IV^poat(O)]^−^ [1,20]. The parallel-mode electron paramagnetic resonance (EPR) spectrum of [Fe^IV^pop(O)]^−^ exhibits a signal at g = 8.2, which supports the assignment of a high-spin, *S* = 2 species (Figure 4B).

**Scheme 2:**
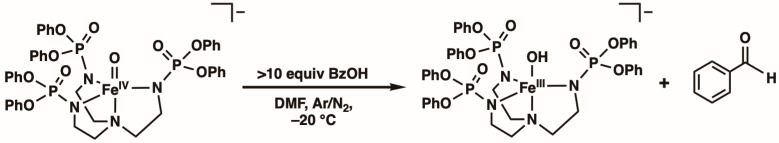
Reactivity of [Fe^IV^pop(O)]^−^ with BzOH in DMF at −20 °C.

**Figure 5:**
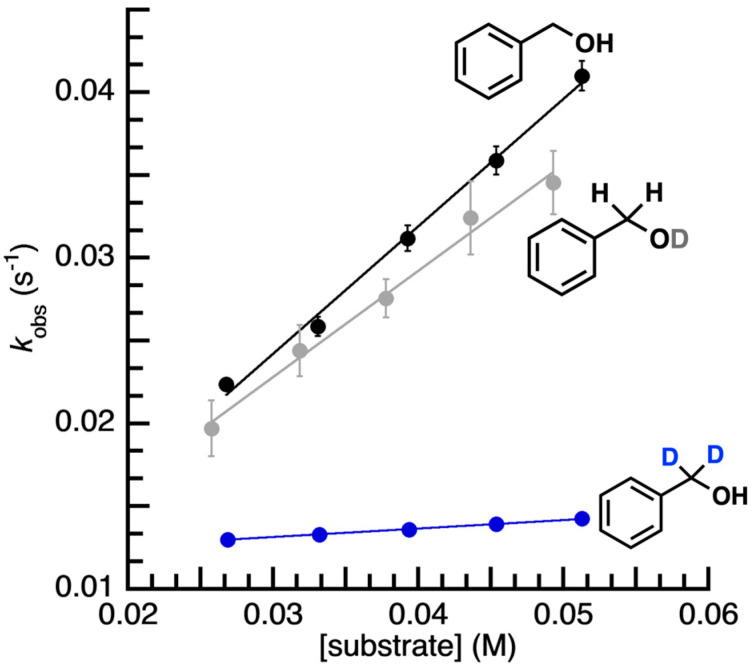
Rate data for [Fe^IV^pop(O)]^−^ + BzOH (black, *k* = 0.193 M^−1^s^−1^), + BzOD (grey, *k* = 0.16 M^−1^s^−1^), and + α,α-*d*_2_-BzOH (blue, *k* = 0.0129 M^−1^s^−1^).

**Reactivity Studies.** The reactivity of [Fe^IV^pop(O)]^−^ with various organic substrates was probed via UV–vis spectroscopy at −20 °C in DMF (no reactivity was found at −80 °C) by monitoring the disappearance of the characteristic *d–d* band associated with the Fe^IV^–oxido unit (see Figure S2). Under these conditions, the self-decay rate of [Fe^IV^pop(O)]^−^ was monitored, and an observed rate constant, *k*_obs_, of 0.012 ± 0.0005 s^−1^ was determined for this process. It was observed that [Fe^IV^pop(O)]^−^ does not react with canonical hydrocarbon substrates such as 9,10-dihydroanthracene, fluorene, xanthene, cumene, or ethyl benzene, among others. We thought that reactivity with external substrates may be promoted if they could participate in H-bonding interactions, which could potentially position the reactive C–H bonds close to the Fe^IV^=O unit. Therefore, we investigated the reactivity with benzyl alcohol (BzOH) because its hydroxyl group could potentially form intermolecular H-bonds with [Fe^IV^pop(O)]^−^. Through spectrophotometric studies, we found that [Fe^IV^pop(O)]^−^ does react with BzOH to yield benzaldehyde and [Fe^III^pop(OH)]^−^ (Scheme 2). The kinetic properties of [Fe^IV^pop(O)]^−^ with BzOH were monitored under pseudo-first-order rate conditions using greater than 10 equiv. of the substrate to obtain *k*_obs_ values for various concentrations of BzOH. A linear correlation was observed between different concentrations of BzOH and the corresponding *k*_obs_ values, whereby *k*_obs_ increased with an increasing concentration of BzOH (Figure 5). From the equation of the line of best fit, the corrected second-order rate constant, *k*, for the reaction of [Fe^IV^pop(O)]^−^ with BzOH was determined to be 0.193 M^−1 ^s^−1^, which accounts for the 2 chemically equivalent C–H bonds per substrate molecule (Figure S3). Experiments in which the substrate concentration was kept constant over the course of the reaction while varying the concentration of [Fe^IV^pop(O)]^−^ were also performed to show that the rate of the reaction was second-order (Figure S4).

To further probe the reactivity of [Fe^IV^pop(O)]^−^ with BzOH, we investigated the reactivity of various *para*-substituted benzyl alcohols and found an increase in the apparent rate constants determined under pseudo-first-order conditions for substrates with more electron-withdrawing substituents (Figure S5A). However, while a Hammett analysis of these substrates led to a linear trend (Figure S5B), the results are complicated by the observation that the y-intercepts extrapolated from the lines of best fit for the *k*_obs_ v. [R-BzOH] of each substrate are not the same, nor do they match the value of the self-decay rate constant of [Fe^IV^pop(O)]^−^. These results support the idea that a pre-equilibrium complex may be formed prior to reactivity (Scheme S1). One consequence of this model is that the addition of a substrate shifts the speciation of the reaction mixture towards a pre-equilibrium complex whose stability is dictated by the nature of the substrate. Assuming that the H-bond donation of R-BzOH derivatives is the predominant binding interaction in the formation of a complex of [Fe^IV^pop(O)]^−^ with the substrate preceding C–H reactivity, then any equilibrium binding constants for these reactants will vary accordingly. As *k*_obs_ is a function of both the C–H reaction rate and the equilibrium constant for the {[Fe^IV^pop(O)]^−^•substrate} complexes, the qualitative results of these experiments support the assignment of a mechanism that involves an association step that is, in part, controlled by the H-bond strength of the substrate.

Assuming the above possible mechanism is operative, the equilibrium binding constant is expected to be relatively weak (i.e., close to one) for BzOH based on the results of kinetic isotope effect (KIE) studies. When α,α-*d*_2_-BzOH was used as the substrate, we found the value of *k*_obs_ for different concentrations of α,α-*d*_2_-BzOH to be smaller than for the undeuterated substrate (Figure 5). In addition, the slope of the line for *k*_obs_ versus increasing concentrations of α,α-*d*_2_-BzOH was smaller, and the y-intercept of the line of best fit gave a *k_obs_* value that was equal to the self-decay rate constant for [Fe^IV^pop(O)]^−^ (*k*_obs_ *=* 0.012 s^−1^). This finding suggests that any equilibrium binding is weak and, therefore, the concentration to reach saturating conditions would be higher than we can obtain. While the reaction is clearly slowed for the α,α-*d*_2_-BzOH substrate, suggesting that the benzylic C–H/D cleavage is rate-limiting, the corresponding KIE value is ill-defined for the substrates investigated. We note that there was no substantial rate change for the reaction with BzOD, benzyl alcohol in which deuteration was solely at the hydroxyl position. A second-order rate constant of 0.16 M^−1^s^−1^ was measured for the reaction of [Fe^IV^pop(O)]^−^ with BzOD, giving a KIE of 1.2, indicating that the breaking of the O–H bond is not involved in the rate-determining step.

**Figure 6:**
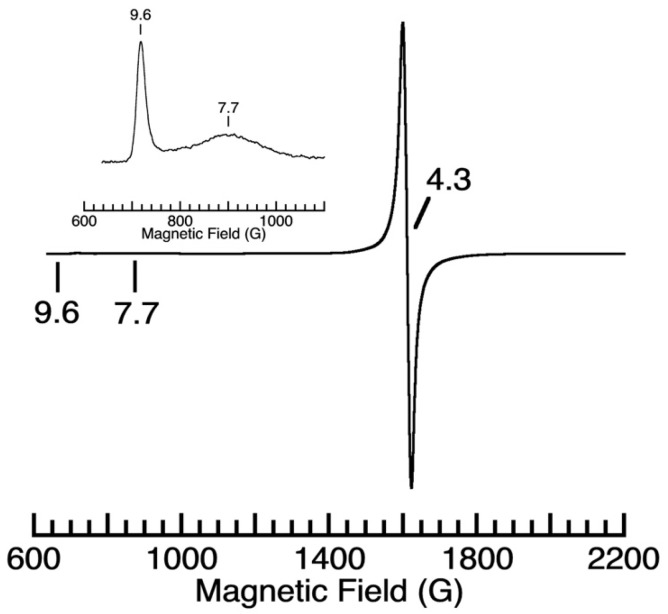
Perpendicular-mode EPR spectrum of the inorganic product from the reaction of [Fe^IV^pop(O)]^−^ in the presence of 9.89 equiv. of benzyl alcohol in DMF:THF collected at 8.2 K. The inset contains an expanded view of the low-field region.

The reaction studies via gas chromatography/mass spectrometry (GCMS) and the ^1^H nuclear magnetic resonance (NMR) experiments involving a bulk reaction of one equiv. of [Fe^IV^pop(O)]^−^ and one equiv. of BzOH support the assignment of benzaldehyde as the organic product (Figure S6 and S7, respectively). For instance, the ^1^H NMR spectrum of the extracted organic product showed a peak at 10.02 ppm, which is consistent with the aldehydic proton from benzaldehyde. In addition, these studies showed that BzOH remains in an approximately 1:1 ratio with benzaldehyde, suggesting that the stoichiometry of the reaction is 2 [Fe^IV^pop(O)]^−^ complexes for every 1 BzOH molecule. This result is reasonable considering that the putative mechanism for this transformation also involves the cleavage of the O–H bond. The EPR spectrum of the solution obtained after the completion of the reaction showed signals at g-values of 9.6, 7.7, and 4.3, which are suggestive of a Fe^III^–hydroxido complex, [Fe^III^pop(OH)]^−^, as the inorganic product (Figure 6). Moreover, [Fe^IV^pop(O)]^−^ does not react with benzyl methyl ether (BzOMe), suggesting that the hydroxyl group is important for function (Figure S8).

*Mechanistic Considerations.* Most reported Fe^IV^–oxido complexes react with external substrates to cleave C–H bonds, especially those with *S* = 2 spin states. Our high-spin Fe^IV^–oxido complexes are the exceptions with both the previously reported [Fe^IV^H_3_buea(O)]^−^ ([H_3_buea]^3−^, tris[(*N*’-*tert*-butylureaylato)-*N*-ethylene]aminato), and [Fe^IV^poat(O)]^−^, which are unreactive toward C–H bonds at –60 °C. We have argued that the intramolecular H-bonds within [Fe^IV^H_3_buea(O)]^−^ and the constrained secondary coordination sphere in [Fe^IV^poat(O)]^−^ hinder reactivity with external substrates. [Fe^IV^pop(O)]^−^ appears to follow a similar trend in that there is no reactivity with most substrates. However, only in the presence of a hydroxyl group at the benzylic position is reactivity observed, which leads us to suggest that there is a pre-association that could be promoted via *intermolecular* H-bonding interaction(s) between the complex and the substrate prior to C–H bond cleavage. This premise is supported by the lack of reactivity observed when BzOMe was used as the substrate. However, just having phosphoryl amido groups appears to not be enough to promote reactivity. We monitored the reaction of [Fe^IV^poat(O)]^−^ with BzOH under the same experimental conditions used with [Fe^IV^pop(O)]^−^ (Figure S9), and no reactivity was observed. It thus seems that the added flexibility of the [pop]^3−^ ligand caused by the ether linkages may provide more accessibility for BzOH to interact. We point out that [Fe^IV^pop(O)]^−^ has a slightly larger E/D value of 0.04 than [Fe^IV^poat(O)]^−^ (0.014) and [Fe^IV^H_3_buea(O)]^−^ (0.03). Thus, both these structural features may be necessary for reactivity.

## 3. Summary

The Fe^IV^–oxido complex, [Fe^IV^pop(O)]^−^, represents a non-heme iron system that oxidizes BzOH to benzaldehyde. The C_3_ symmetry in conjunction with the trianionic equatorial ligand field helps enforce a high-spin state at the Fe^IV^ center, a property that is thought to be a key factor in C–H bond cleavage by Fe^IV^–oxido moieties in metalloenzymes. The K[Fe^II^pop(OH_2_)] salt in the presence of a crown ether acts as a reliable synthon for the generation of the corresponding Fe^IV^–oxido complex, whose spectroscopic properties are consistent with this formulation. The studies found that [Fe^IV^pop(O)]^−^ is unreactive toward a variety of external substrates bearing C–H bonds, apart from BzOH. In addition, KIE studies with deuterated benzyl alcohol derivatives indicated that cleavage of a benzylic C–H bond is involved in the rate-determining step. We abstain from making precise statements regarding the mechanism based on the available data; however, it appears that the structural properties of [Fe^IV^pop(O)]^−^ contribute to this function. The ether linkages within the phosphoryl amido groups produce a more flexible cavity around the Fe^IV^=O unit to increase accessibility, and it is likely that weak intermolecular H-bond(s) between BzOH and [Fe^IV^pop(O)]^−^ are formed prior to C–H bond cleavage. Therefore, the coupling of H-bond formation with a less constrained secondary coordination sphere could lead to the observed conversion of BzOH to benzaldehyde, illustrating the role of the secondary coordination sphere in metal-ion-mediated processes.

## 4. Experimental Section

### 4.1. General Procedures

All reactions were performed under an argon or nitrogen atmosphere in a Vacuum Atmospheres Company (VAC) dry box or on a Schlenk line unless otherwise stated. Reagents were used as received from commercial sources unless otherwise stated. Solvents were sparged with argon and dried over columns containing Q-5 and molecular sieves. DMF was either sparged with argon and dried over columns containing Q-5 and molecular sieves or purchased from Sigma Aldrich. Potassium hydride as a 30% suspension in mineral oil was filtered and washed with Et_2_O (5 × 10 mL) and pentane (5 × 10 mL), dried under reduced pressure, and stored in a dry box at room temperature. Triethylamine was vacuum-distilled with gentle heating prior to being used for the ligand synthesis. Fe^II^(OAc)_2_ [25] and IBX-iPr [26,27] were synthesized according to the procedures in the literature. 4-Cl-benzyl alcohol and 4-Me-benzyl alcohol were recrystallized with ethanol multiple times prior to use.

### 4.2. Physical Methods

Electronic absorption spectra were collected in a 1 cm quartz cuvette containing a magnetic stir bar inside. Kinetic measurements were recorded with an 8453E Agilent UV-vis spectrometer sourced from Agilent Technologies, Santa Clara, CA, USA, equipped with a Unisoku Unispeks cryostat sourced from Unisoku Co., Ltd. in Hirakata City, Osaka, Japan. X-band EPR measurements were recorded using a Bruker EMX spectrometer from Bruker Scientific, Billerica, MA USA equipped with an ER041XG microwave bridge, an Oxford liquid-helium quartz cryostat from Oxford Scientific, Oxford, UK. A modulation frequency of 100 kHz was used for all EPR spectra. ^1^H NMR experiments were measured using either a 600 MHz Bruker AVANCE spectrometer from Bruker Scientific, Billerica, MA USA with a BBFO (broadband fluorene observe) cryoprobe as a standard, a 500 MHz Bruker DRX spectrometer with a TCI (three-channel inverse) cryoprobe as a standard, or a 400 MHz Bruker DRX spectrometer with a switchable QNP (quad-nucleus probe) cryoprobe as a standard. Both instruments are from Bruker Scientific, Billerica, MA USA. GCMS data were measured using a Waters Micromass GCT Premier mass spectrometer from Waters Corporation, Milford, MA USA with a DB-5 30 m × 0.25 mm × 0.25 µ film column, helium as the carrier gas, and the positive Cl (ammonia reagent gas) mode for ionization. Solid-state FTIR spectra were collected using a Thermo Scientific Nicolet iS5 FT-IR spectrometer (Waltham, MA USA) equipped with an iD5 ATR accessory.

### 4.3. Preparative Methods

*H_3_pop*. To a clear, colorless solution of tris(2-aminoethyl)amine (tren) (4.525 g, 30.94 mmol) and triethylamine (46.84 g, 462.9 mmol) in 200 mL of tetrahydrofuran (THF) in a round-bottom flask, diphenyl phosphoryl chloride (25.0 g, 93.1 mmol) in 80 mL of THF was added dropwise while stirring. The reaction mixture immediately formed into a white and cloudy heterogeneous solution. Once the addition of diphenyl phosphoryl chloride was complete, 20 mL of THF was added to rinse the addition funnel. The addition funnel was then removed, the round-bottom flask was covered with a glass stopper, and the reaction mixture was left to stir overnight. The flask was brought to ambient conditions the next day for workup. The white precipitate (Et_3_NHCl) was filtered off, and the solvent was removed until a thick oil was observed. Diethyl ether (Et_2_O; 200 mL) was added to the resulting oil and stirred vigorously over 30–60 min to slowly precipitate out a white powder (24.72 g, 94.70%). The white powder was collected on a medium porosity glass-fritted funnel, washed with Et_2_O (5 × 20 mL), and dried for several hours under reduced pressure. The dried, white solid was placed into a glovebox and stored under an Ar atmosphere. ^1^H NMR (500 MHz, *d*_6_-DMSO): δ 2.50 (t, 6H), 3.11 (q, 6H), 6.13 (q, 3H), 7.45 (t, 12 H), 7.50 (d, 6H), and 7.61 (t, 12 H); ^13^C NMR (125 MHz, CDCl_3_, ppm): 151.0, 130.3, 125.2, 120.5, 56.5, and 47.2; ^31^P NMR (162 MHz, CDCl_3_): 21.9; FTIR (Diamond ATR, cm^−1^): 3300 (N–H, sh), 3232 (N–H), 3064, 3042, 2945, 2877, 2833, 2805, 2600, 1588, 1485, 1455, 1440, 1263, 1240, 1213, 1188, 1162, 1108, 1070, 1053, 1024, 1007, 975, 917, 830, 807, 759, 688, and 616. HRMS (ES+, m/z): the exact mass calculated for NaC_42_H_45_N_4_O_9_P_3_ [M + Na] was 865.23; found: 865.2297.

*K[Fe^II^pop(OH_2_)]*. To a clear, colorless solution of H_3_pop (0.4009 g, 0.4757 mmol) in 6 mL of anhydrous THF, 3 equiv. of KH (0.0575 g, 1.43 mmol) was added. The reaction mixture was allowed to stir for at least one hour or until H_2_ evolution ceased. Once deprotonation of the amide nitrogens was complete, 1 equiv. of Fe^II^(OAc)_2_ (0.0828 g, 0.476 mmol) was added, resulting in an immediate change to an orange, heterogeneous solution. After approximately 1 h of stirring, 1 equiv. of H_2_O (10 µL, 0.55 mmol) was added via syringe, resulting in an immediate change to an off-white, heterogeneous solution. The reaction mixture was allowed to stir for an additional 10 min and the KOAc byproduct (0.0793 g, 84.92%) was then filtered off using a medium-porosity frit. The resulting clear, pale yellow solution was layered under pentane to yield colorless crystals suitable for XRD methods (0.384 g, 85.0%). UV–vis (DMF:THF, −80 °C): λ_max_/nm (ε/M^−1^cm^−1^) = 960 (4.7). FTIR (Diamond ATR, cm^−1^): 3226 (O–H), 3064, 3042, 2955, 2892, 2852, 2683, 1588, 1485, 1263, 1240, 1229, 1195, 1154, 1123, 1070, 1045, 1024, 1004, 987, 908, 882, 819, 761, and 688. Elemental analysis calcd. for C_42_H_44_FeKN_4_O_10_P_3_•0.5 H_2_O: C, 52.46; H, 4.72; and N, 5.83%. Found: C, 52.30; H, 4.77; and N, 5.76%.

*Low-Temperature Solution Studies of [Fe^IV^pop(O)]^−^.* For the UV–vis studies, the generation of [Fe^IV^pop(O)]^−^ was performed in a cuvette under an Ar atmosphere at −80 °C in a solution of 1:1 DMF:THF. The Fe^II^ stock solution was prepared by dissolving crystalline material of either K[Fe^II^pop] or K[Fe^II^pop(OH)_2_] as the starting complex in the presence of at least 2 equiv. of 18c6. A cuvette filled with 2 mL of 1:1 DMF:THF was sealed with a rubber septum inside a glove box, transferred into the cryostat of a spectrophotometer, and then allowed to equilibrate at −80 °C for approximately 15 min. Using a gastight syringe, an aliquot of the Fe^II^ stock solution was injected into the cuvette and mixed for ~150 s, after which an aliquot of the IBX-iPr stock solution was injected to generate [Fe^IV^pop(O)]^−^. For the EPR studies, the generation of [Fe^IV^pop(O)]^−^ was achieved by first preparing a solution of IBX-iPr in 1:1 DMF:THF in an EPR tube sealed with a rubber septum inside a glove box. The EPR tube was taken out of the glove box, placed into a dry ice/acetone cold bath at −80 °C, and allowed to equilibrate for 30 min. Then, an aliquot of a stock solution of K[Fe^II^pop(OH_2_)] in the presence of 2.25 equiv. of 18c6 was added to the EPR tube, mixed every 2 min for a total of 20 min, and then froze in liquid nitrogen.

*Kinetic Measurements.* All kinetic measurements were performed under an Ar or N_2_ atmosphere in anhydrous DMF at −20 °C in a 1 cm pathlength cuvette equipped with a magnetic stir bar. In a typical kinetic experiment, stock solutions of ~68 mM K[Fe^II^pop(OH_2_)] + 2 − 2.5 equiv. of 18c6 (K(18c6)_2_[Fe^II^pop(OH_2_)]), ~53 mM IBX-iPr, and ~580 mM substrate were prepared at room temperature. A cuvette was filled with 2 mL of DMF and sealed with a rubber septum, and was then taken out of the glove box and transferred into the cryostat of the spectrophotometer. The cuvette was allowed to equilibrate at the desired temperature for approximately 15 min, and then 60 µL (0.0041 mmol) of the K(18c6)_2_[Fe^II^pop(OH_2_)] stock solution was injected into the cuvette using a gastight syringe to reach a desired concentration of ~2 mM. The mixture was then treated with 80 µL (0.0042 mmol) of a 53 mM stock solution of IBX-iPr to generate K(18c6)_2_[Fe^IV^pop(O)]. Immediately after the formation of the 900 nm band associated with [Fe^IV^pop(O)]^−^ reached its maximum absorbance (t = 20–30 s), 100−200 µL of the substrate (> 10 equiv.) was added via a gastight syringe. The reaction progress was measured by monitoring the decrease in the 900 nm band over time (in seconds). Once the reaction was complete, the color of the solution in the cuvette was pale yellow.

Kinetic measurements for each concentration of substrate were repeated at least 3 times. The expression ln[(A_t_–_f_)/(A_i_–A_f_)] was plotted against the reaction time for the first three half-lives to produce a linear correlation. In this expression, A_t_ is the absorbance at time t, A_i_ is the initial absorbance, and A_f_ is the final absorbance of the band at 900 nm. In the plot of ln[(A_t_–A_f_)/(A_i_–A_f_)] vs. t, the slope of the line divided by 2 corresponds to the observed rate constant, *k*_obs_ (s^−1^). The division by 2 is necessary to account for the overall stoichiometry of the reaction, i.e., for every oxidation of 1 benzyl alcohol molecule, 2 equiv. of Fe^IV^ complexes were consumed.

*Preparation of BzOD for KIE Studies.* In a round-bottom flask, approximately 4 mL of BzOH was combined with 2.6 mL of D_2_O and vigorously stirred. The product was extracted 3 times with 10 mL aliquots of Et_2_O. The Et_2_O was added directly to the round-bottom flask and shaken. The resulting two layers were allowed to separate. The Et_2_O layer was pipetted off, dried over Na_2_SO_4_, filtered into a Schlenk flask, and dried under reduced pressure. ^1^H NMR (500 MHz, CDCl_3_): δ 4.71 (s, 2H) and 7.30–7.37 (aromatics, 5H).

*GC-MS and ^1^H NMR Experiments for the Organic Products.* Separate solutions of IBX-iPr (10.6 mg, 0.0329 mmol) in 2 mL of DMF and K[Fe^II^pop] (33.8 mg, 0.0362 mmol) + 18c6 (16.7 mg, 0.0632 mmol) in 0.1 mL of DMF were stored in aluminum blocks in a freezer at –30 °C for approximately 30 min. The IBX-iPr solutions were then taken out of the freezer and placed on a stir plate while still remaining in the aluminum block. Afterward, the pre-chilled K[Fe^II^pop] + 18c6 solution was quickly added to the IBX-iPr solution and stirred for approximately 10 min, changing from red to orange. The organic compound in the reaction mixture was extracted 3 times with 2–3 mL of pentane, after which the solvent was removed under reduced pressure to produce a colorless liquid. Half of the resulting liquid was dissolved in 1 mL of CH_2_Cl_2_ and diluted 30-fold for GC-MS measurements, and the other half was dissolved in 750 µL of *d*_6_-DMSO for ^1^H NMR measurements.

### 4.4. X-ray Crystallographic Methods

For {K[Fe^II^pop(OH_2_)]}_2_, a colorless crystal with approximate dimensions of 0.379 × 0.338 × 0.152 mm was mounted in a cryoloop and transferred to a Bruker SMART APEX II diffractometer from Bruker Scientific, Billerica, MA USA. The APEX2 program package was used to determine the unit cell parameters and for data collection (with a 30 s/frame scan time for the sphere of diffraction data). The raw frame data were processed using SAINT and SADABS to yield the reflection data file. Subsequent calculations were carried out using the SHELXTL program. There were no systematic absences nor any diffraction symmetry other than the Friedel condition. The centrosymmetric triclinic space group *P*$\overset{–}{1}$ was assigned and later determined to be correct. The structure was solved using direct methods and refined on F^2^ employing full-matrix least-squares techniques. The analytical scattering factors for neutral atoms were used throughout the analysis. Hydrogen atoms (H1A, H1B, H11A, and H11B) on O1 and O11 of the aqua ligands were located from a difference Fourier map and refined (x, y, z, and U_iso_). The remaining hydrogen atoms were included using a riding model. The least-squares technique yielded wR2 = 0.1080 and Goof = 1.038 for 1115 variables refined against 20941 data (0.74 Å), and R1 = 0.0392 for those 16,759 data with I > 2.0σ(I).

## Data Availability

The data presented in this study are available within this article and the accompany Supplementary Materials.

## Supplementary Materials

The following supporting information can be downloaded at: https://www.mdpi.com/article/10.3390/molecules28124755/s1, Figure S1: Zoomed in electronic absorption spectrum of [Fe^IV^pop(O)]^−^; Figure S2: Electronic absorption spectrum of the reaction of [Fe^IV^pop(O)]^−^ with BzOH and the corresponding pseudo first-order kinetic plots.; Figure S3: Plot of *k*_obs_ vs. the concentration of BzOH.; Figure S4: Plot of *k* vs. the concentration of [Fe^IV^pop(O)]^−^.; Figure S5: Plot of *k*_obs_ vs. the concentration of 4-R-BzOH and the corresponding Hammett plot.; Figure S6: GCMS data for the reaction of [Fe^IV^pop(O)]^−^ with BzOH (1:1).; Figure S7: ^1^H NMR spectra comparing benzaldehyde to the extracted organic product from the reaction of [Fe^IV^pop(O)]^−^ with and without BzOH.; Figure S8: Plot of *k*_obs_ vs. the concentration of BzOMe.; Figure S9: Plot of *k*_obs_ vs. the concentration of BzOH using either [Fe^IV^pop(O)]^−^ or [Fe^IV^poat(O)]^−^ as the oxidant.; Scheme S1: Expression for the proposed pre-equilibrium between [Fe^IV^pop(O)]^−^ and substrate.; Table S1: Second order rate constants and the corresponding substituent constant for *para*-substituted benzyl alcohols.; Table S2: Crystallographic information for {K[Fe^II^pop(OH_2_)]}_2_.
