# Supplementary material for: Selective C–H Bond Cleavage with a High-Spin FeIV–Oxido Complex
Source: Molecules. 2023 Jun 14;28(12):4755. doi: 10.3390/molecules28124755 (PMC10301140; doi:10.3390/molecules28124755)
Supplement: Supplementary file 1 [file molecules-28-04755-s001.zip › molecules-2306291-supplementary.pdf]

Supporting Information for

Selective C–H Bond Cleavage by a High Spin Fe^{IV}–Oxido Complex

Chen Sun, Jennifer L. Jaimes, Alec H. Follmer, Joseph W. Ziller, and A.S. Borovik*

Table of Contents

Figure S1. Zoomed in electronic absorption spectrum of $[Fe^{IV}pop(O)]^{-}$. S2

Figure S2. Electronic absorption spectrum of the reaction of $[Fe^{IV}pop(O)]^{-}$ with BzOH and the corresponding pseudo first-order kinetic plots. S2

Figure S3. Plot of k_{obs} vs. the concentration of BzOH. S3

Figure S4. Plot of k_{obs} vs. the concentration of $[Fe^{IV}pop(O)]^{-}$. S3

Figure S5. Plot of k_{obs} vs. the concentration of 4-R-BzOH and the corresponding Hammett plot. S4

Figure S6. GCMS data for the reaction of $[Fe^{IV}pop(O)]^{-}$ with BzOH (1:1). S4

Figure S7. ^{1}H NMR spectra comparing benzaldehyde to the extracted organic S4 product from the reaction of $[Fe^{IV}pop(O)]^{-}$ with and without BzOH. S5

Figure S8. Plot of k_{obs} vs. the concentration of BzOMe. S5

Figure S9. Plot of k_{obs} vs. the concentration of BzOH using either $[Fe^{IV}pop(O)]^{-}$ or $[Fe^{IV}poat(O)]^{-}$ as the oxidant. S6

Scheme S1. Expression for the proposed pre-equilibrium between $[Fe^{IV}pop(O)]^{-}$ and substrate. S6

Table S1. Second order rate constants and the corresponding substituent constant for *para*-substituted benzyl alcohols. S7

Table S2. Crystallographic Information for $\{K[Fe^{II}pop(OH_2)]\}_2$. S7

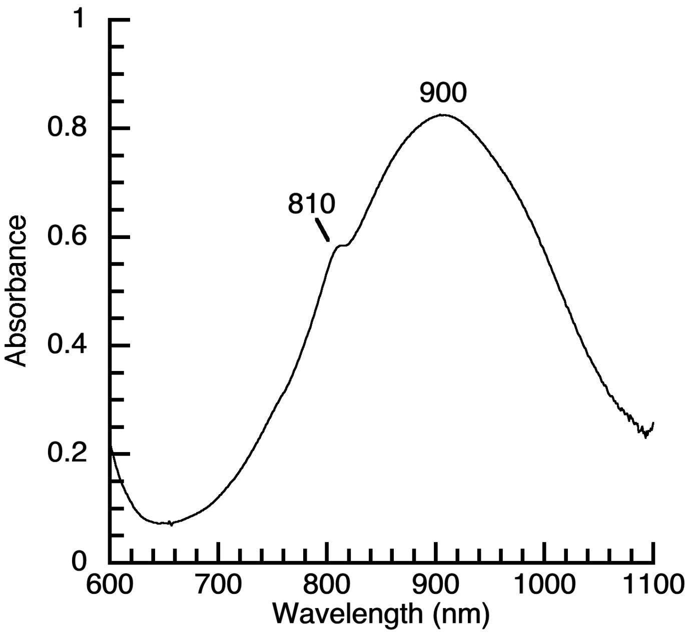

Figure S1. Electronic absorption spectrum of $[Fe^{IV}pop(O)]^-$ generated using 9.23 mM $K[Fe^{II}pop(OH_2)]$ in the presence of 2.08 equiv of 18c6 + 1.06 equiv of IBX-iPr in DMF:THF at –80 ºC, highlighting the low-energy region.

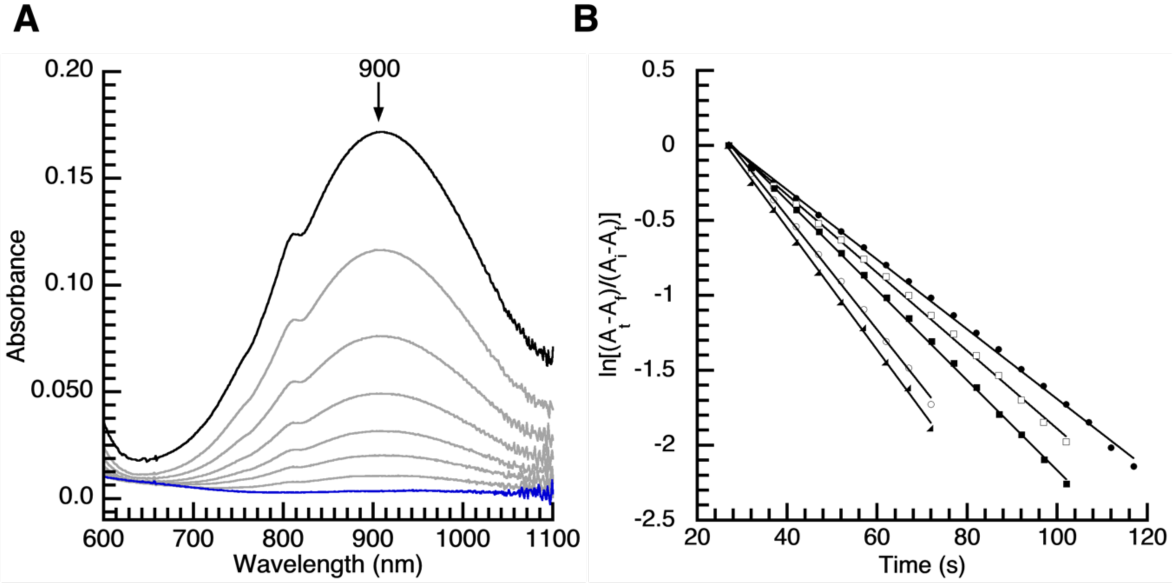

Figure S2. (A) Electronic absorption spectrum showing the disappearance of the 900 nm *d–d* band from the reaction of $[Fe^{IV}pop(O)]^-$ (1.9 mM) and BzOH under pseudo first-order rate conditions in DMF at –20 ºC. (B) The corresponding plot of $\ln\left(\frac{A_t-A_f}{A_i-A_f}\right)$ vs. time associated with the disappearance of the 900 nm *d–d* band for different concentrations of BzOH.

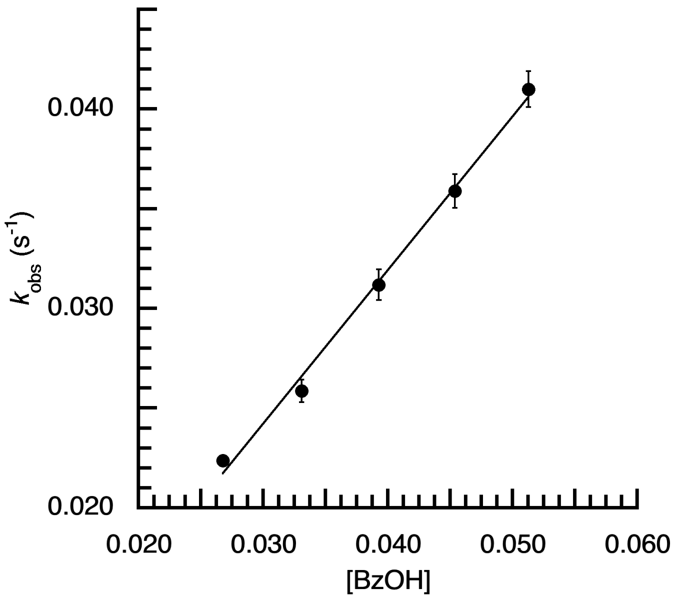

Figure S3. Plot of k_{obs} vs. the concentration of benzyl alcohol in DMF at –20 °C.

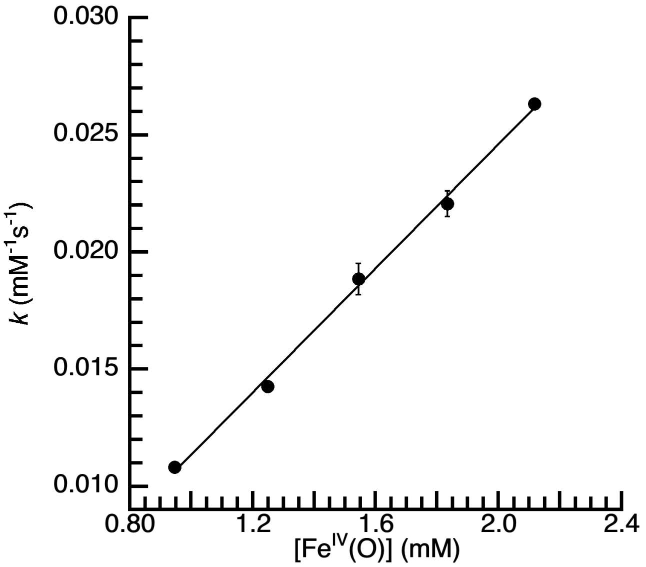

Figure S4. Plot of k vs. the concentration of [Fe^{IV}pop(O)]$^{-}$ in DMF at –20 °C.

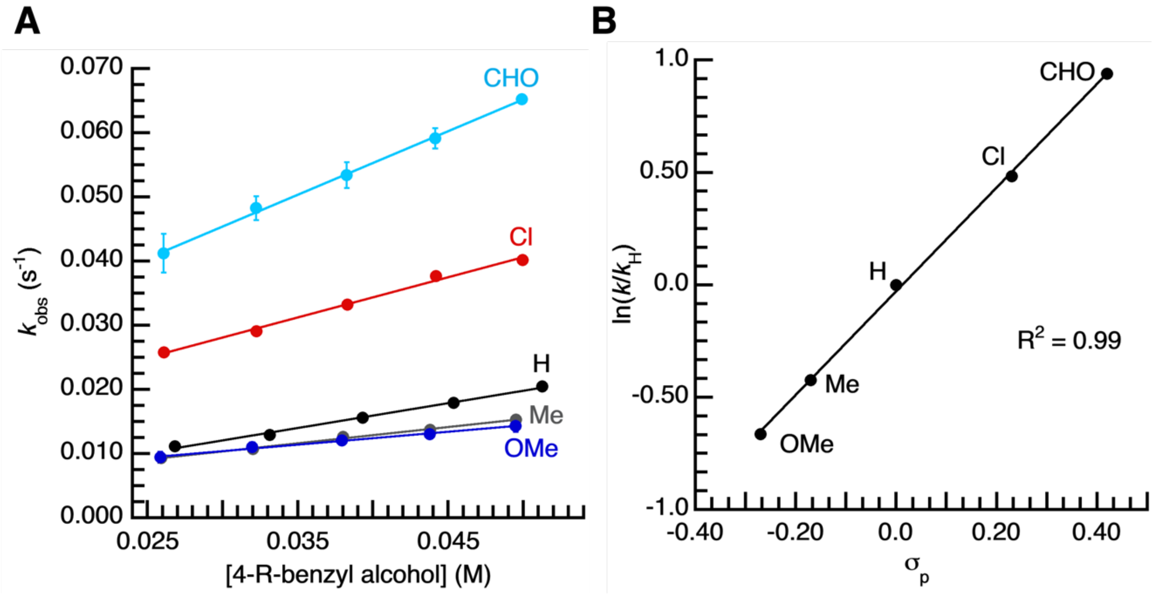

Figure S5: (A) Plot of k_{obs} vs. the concentration of 4-R-BzOH in DMF at –20 °C and (B) the corresponding Hammett plot.

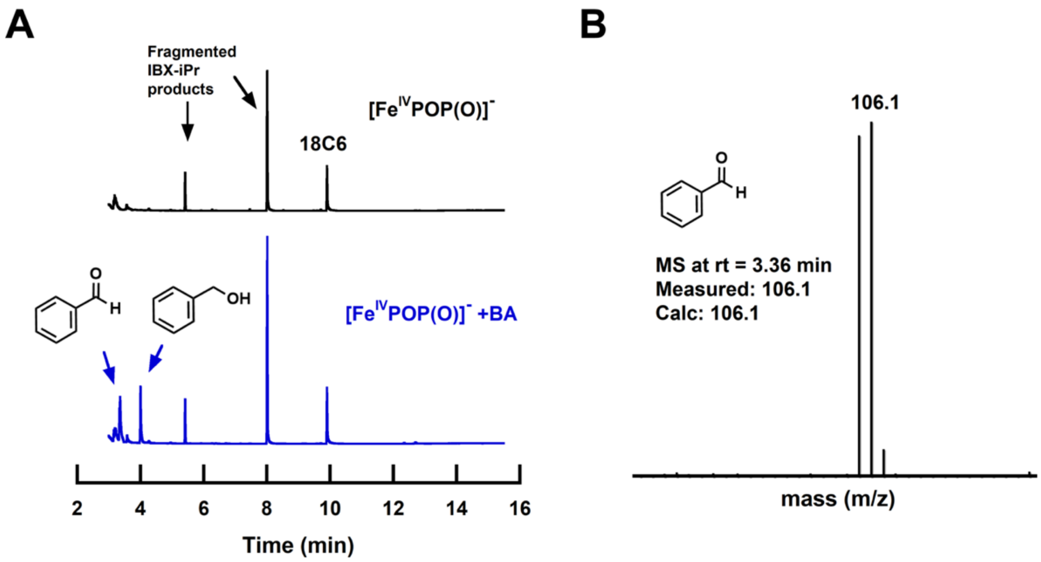

Figure S6. (A) Gas chromatograph of the organic products from the reaction of [Fe^{IV}pop(O)]$^-$ with BzOH (1:1) and (B) the corresponding mass spectrum at retention time (rt) 3.36 min in CH_2Cl_2.

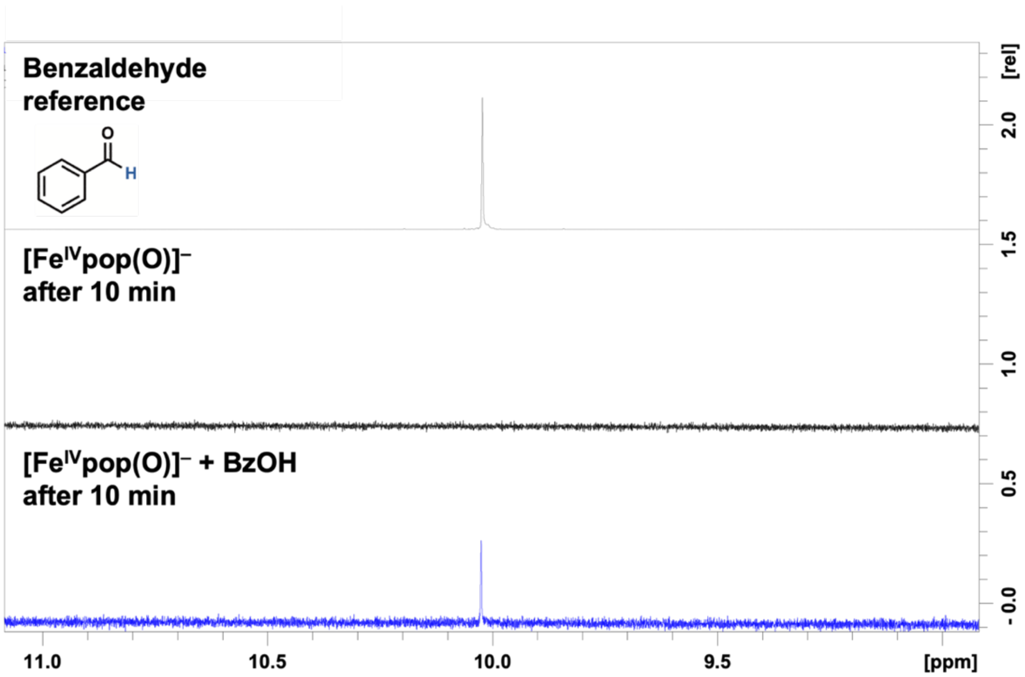

Figure S7. ^{1}H NMR spectrum in d_6-DMSO of an authentic sample of benzaldehyde (grey), the extracted organic product from the reaction of [Fe^{IV}pop(O)]$^{-}$ without BzOH (black), and the extracted organic product from the reaction of [Fe^{IV}pop(O)]$^{-}$ with BzOH (1:1) (blue).

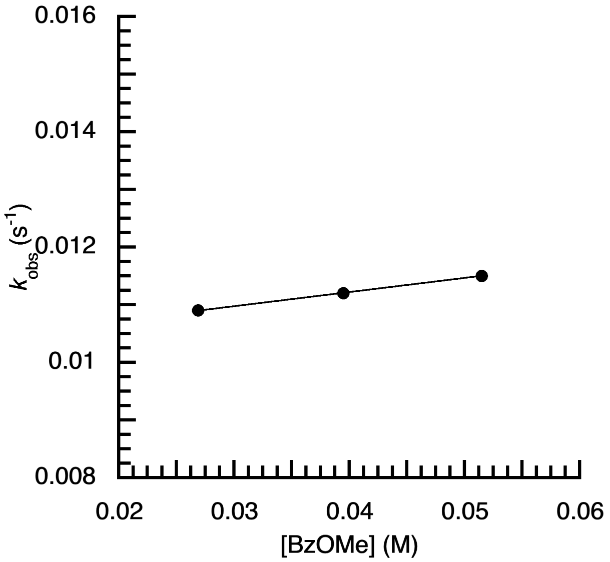

Figure S8. Plot of k_{obs} vs. the concentration of BzOMe in DMF at –20 ºC.

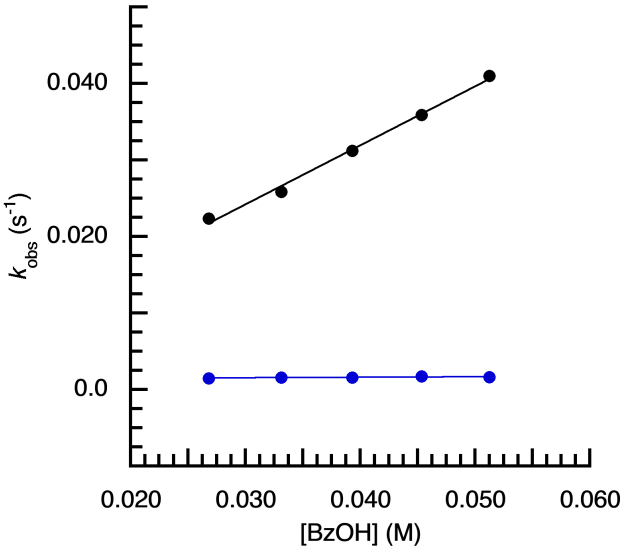

Figure S9. Plot of k_{obs} vs. the concentration of BzOH in DMF at –20 °C using either [Fe^{IV}pop(O)]$^{–}$ (black) or [Fe^{IV}pop(O)]$^{-}$ or [Fe^{IV}poat(O)]$^{-}$ (blue) as the oxidant.

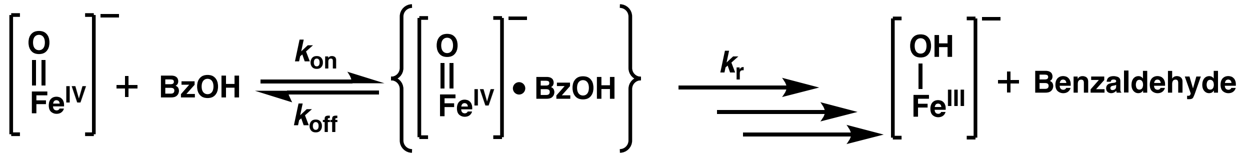

Scheme S1. Expression for the proposed pre-equilibrium between the Fe^{IV}–oxido complex and substrate.

Table S1. Second order rate constants (k) and the corresponding substituent constant (σ) for each respective *para*-substituted benzyl alcohol.

**R**	**k ($M^{-1}s^{-1}$)**	**σ**
OMe	0.100	-0.27
Me	0.127	-0.17
H	0.193	0
Cl	0.314	0.23
CHO	0.495	0.42

Table S2. Crystallographic Information for $\{K[Fe^{II}pop(OH_2)]\}_2$

**Complex**	**$\{K[Fe^{II}pop(OH_2)]\}$**
Empirical Formula	$C_{84}H_{86}Fe_2K_2N_8O_{20}P_6$
Fw	1903.32
T (K)	133(2)
Crystal System	Triclinic
Space Group	$P\bar{1}$
a (Å)	14.427(3)
b (Å)	16.428(3)
c (Å)	20.699(4)
α (°)	99.145(2)
β (°)	98.055(2)
γ (°)	111.423(2)
V ($Å^3$)	4402.8(13)
Z	2
δ_{calcd} (Mg/m^3)	1.436
GOF on F^2	1.038
R1	0.0392
wR2	0.1080
